# Cod-Liver Oil

**Published:** 1849

**Authors:** 


### Article X.—*Cod-Liver Oil.*

A discussion on the properties of this article took place at the Westminster Medical Society, February 3. The majority of the fellows stated that they had found the oil to possess a very marked effect in almost all cases of scrofula and phthisis. In the first class of cases, it was not only given internally, with the effect of much improving the general health, bur it was applied locally to scrofulous sores, with the most marked benefit. In phthisis it appeared to exert its influence at once by its nutritious properties. It checked perspiration, and removed emaciation; and appeared, by keeping up the tone of the system, to arrest the further deposition of tubercular matter. Some thought that any oily substance, as butter or almond oil, would have the same effect; others considered the cod-liver oil to have some specific influence. One gentleman had found it rather injurious than otherwise in some cases of phthisis, from its tendency to disorder the digestive organs. Altogether, however, the opinion generally was decidedly in its favor as a palliative agent in consumption.—*Lancet* in *Med. News.*

